# Recent Advances in the Mechanisms and Regulation of QS in Dairy Spoilage by *Pseudomonas* spp.

**DOI:** 10.3390/foods10123088

**Published:** 2021-12-13

**Authors:** Laura Quintieri, Leonardo Caputo, Milena Brasca, Francesca Fanelli

**Affiliations:** 1 Institute of Sciences of Food Production, National Research Council of Italy, 70126 Bari, Italy; leonardo.caputo@ispa.cnr.it (L.C.); francesca.fanelli@ispa.cnr.it (F.F.); 2 Institute of Sciences of Food Production, National Research Council of Italy, 20133 Milan, Italy; milena.brasca@ispa.cnr.it

**Keywords:** pseudomonads, dairy products, spoilage traits, biofilm, *quorum sensing*, metabolic pathways, QS inhibitors

## Abstract

Food spoilage is a serious issue dramatically impacting the worldwide need to counteract food insecurity. Despite the very expensive application of low temperatures, the proper conservation of fresh dairy products is continuously threatened at different stages of production and commercialization by psychrotrophic populations mainly belonging to the *Pseudomonas* genus. These bacteria cause discolouration, loss of structure, and off-flavours, with fatal implications on the quality and shelf-life of products. While the effects of pseudomonad decay have been widely reported, the mechanisms responsible for the activation and regulation of spoilage pathways are still poorly explored. Recently, molecule signals and regulators involved in *quorum sensing* (QS), such as homoserine lactones, the *lux*R/*lux*I system, *hdtS*, and *psoR,* have been detected in spoiled products and bacterial spoiler species; this evidence suggests the role of bacterial cross talk in dairy spoilage and paves the way towards the search for novel preservation strategies based on QS inhibition. The aim of this review was to investigate the advancements achieved by the application of omic approaches in deciphering the molecular mechanisms controlled by QS systems in pseudomonads, by focusing on the regulators and metabolic pathways responsible for spoilage of fresh dairy products. In addition, due the ability of pseudomonads to quickly spread in the environment as biofilm communities, which may also include pathogenic and multidrug-resistant (MDR) species, the risk derived from the gaps in clearly defined and regulated sanitization actions is underlined.

## 1. Introduction

The increasing global population has contributed to making food insecurity one of the global challenges; in fact, more than 820 million people in the world suffer food scarcity [1], which negatively impacts mental, social, and physical well-being [2]. This crucial issue has been further worsened by the COVID-19 pandemic [3]. Thus, in response to the urgent and increasing global demand for food, several strategies and measures have been proposed by the official authorities [4]; some of these are aimed at reducing food losses and waste at different stages of the food production and supply chains, including the prevention of food loss during processing, the transformation of perishable raw materials into shelf-stable products, the extension of the shelf-life through packaging and processing innovation, the introduction of clear date labels or storage, and the prevention of food spoilage [5]. Spoilage is a deterioration process caused by microbiological, chemical, and physical changes that makes the food product unacceptable for the consumer and causes significant economic losses for the food industry.

In the dairy sector, the application of low temperatures has allowed the extension of the shelf-life and marketing of various fresh dairy products. However, naturally occurring bacteria, such as pseudomonads, have increasingly become a real concern for cold-stored fresh dairy products because of their ability to grow and adapt themselves to low temperatures. They are responsible for visible spoilage traits (discolorations, structure loss, rheology changes) and non-visible defects (protein breakdown, off-odours, and off-flavours), which significantly reduce the quality and shelf-life of dairy products.

*Pseudomonas* spp. contamination routes have been largely studied, especially for milk or milk-based beverages [6,7]. In contrast, how these bacteria manage to contaminate cheeses even made from pasteurized milk is still much debated. Since these are environmental microorganisms generally present in the soil, the main sources of contamination are surfaces, water, and obviously, exposure to air. Pseudomonads contamination occurs at each step of the manufacturing process of dairy products and it becomes more persistent and resistant to sanification procedures when bacterial cells grow as biofilm [8]. In addition, increasing amounts of enzymes (proteases and lipases) and the activation of metabolic pathways correlated to spoilage traits (e.g., pigments biosynthesis) have been found in biofilm rather than in the fluctuating state [9]. Regardless of this evidence, the mechanisms responsible for the production and the activation of spoilage enzymes, metabolites, and pathways are still poorly explored. Most of them are *quorum sensing* (QS) regulated, suggesting its potential role in dairy spoilage [10,11,12]. To confirm this, different molecule signals have been detected in spoiled products, where they affect microbial biodiversity and metabolic activities [13,14]. Thus, the most recent and innovative preservation strategies aim to intercept and inhibit this communication system, rather than to exert antimicrobial activity [12,15]. However, to the best of our knowledge, studies based on the application of QS inhibitors (QSI) to improve the shelf-life of dairy products are performed sporadically [16,17], and most in vitro and in situ studies are focused on pseudomonads isolated from fish [18,19,20]. This knowledge gap highlights the need to explore more deeply the metabolic activities and their regulation of dairy spoilage; this in order to identify the specific markers or novel molecular targets to apply in innovative control strategies, and in so doing reducing food losses and waste in the dairy sector.

Therefore, this review is firstly aimed at deeply analysing the pseudomonads issue in the dairy sector, focusing on the spoilage traits and associated contribution of the transition from a planktonic to biofilm state of bacterial cells. Then, we discuss the QS systems for the most-occurring *Pseudomonas* species, the related spoilage regulators and metabolic pathways, as well as methodological approaches to intercept this complex communication system in dairy products, in order to provide novel insights to be exploited for the development of novel preservation strategies.

## 2. *Pseudomonas* spp. as Major Cause of Spoilage in Dairy Chain

**Figure 1:**
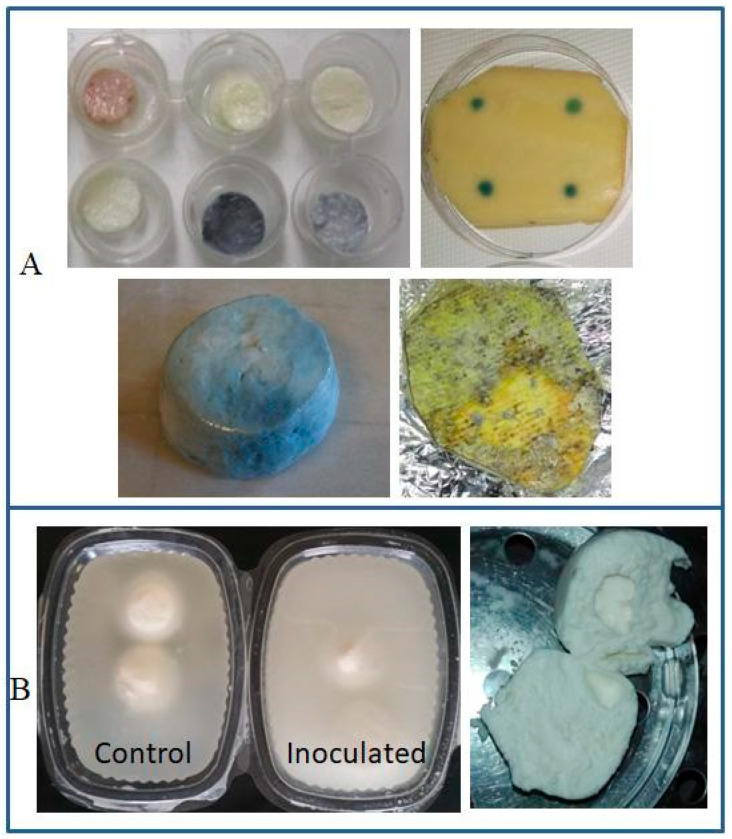
Spoilage traits in dairy products under cold storage conditions: (**A**) discolorations of dairy products contaminated by *Pseudomonas* spp.; (**B**) proteolysis of HM Mozzarella cheeses inoculated with *P. fluorescens* strains (Quintieri L., Caputo L., Brasca M., Fanelli F., unpublished).

Due to their metabolic versatility and adaptation, *Pseudomonas* spp. are widely occurring in dairy products. Although they optimally grow at higher temperatures, some pseudomonad species are also favoured by refrigeration temperatures of fresh foods due to their ability to increase the proportion of unsaturated fatty acids in the lipid phase of their membranes, making them more fluid [21]. This dual ability enables these bacteria to better compete with the natural microbiota of cold-stored fresh foods, and even adapt themselves under inappropriate storage conditions. Unlike other psychrotrophs, pseudomonads show a generation time at 0–7 °C shorter than at the optimal growth temperature [22]. With regard to their need for oxygen, they can grow even in anoxic atmospheres with high percentages of CO_2_ [23]. Due to the high content of nutritional compounds and water and pH neutrality, milk and fresh dairy products are optimal matrices for pseudomonads. The spoilage traits strictly depend on species, food characteristics, storage conditions, and adaptation ability [24]; indeed, *Pseudomonas* species grow and become dominant thanks to the expression of a number of determinants (enzymes, pigments) addressed to enhance competition and adaptation of the bacteria. In this regard, Table 1 and Figure 1 show some of the spoilage traits caused by *Pseudomonas* spp. in dairy products under cold-storage conditions, which are better described in the following sections.

### 2.1. Spoilage Traits Caused by Proteolytic and Lipolytic Activities

In raw milk, psychrotrophic bacteria, such as *P. fluorescens*, *P. fragi*, *P. lactis*, *P. putida,* and *P. gessardii*, are frequent [26,31]. Although these bacteria become inactive after milk pasteurization and sterilization, their extracellular proteases are highly thermostable, and retain the ability to degrade milk proteins, causing bitterness and age gelation in ultra-high temperature (UHT) milk [53]. The proteases released in the milk lead to the extensive breakdown of k-casein to para-k-casein, influencing rennet coagulation [54]. In turn, plasminogen and plasmin are liberated from casein micelles causing additional proteolysis of *α*_S1_-*α*_S2_-casein and *β*-casein, affecting milk texture and flavour [55], but even cheese yield [56]. Among the extracellular proteases produced by *Pseudomonas,* the alkaline zinc metalloprotease AprX retains its activity even after boiling for 10 min, showing a high resistance to heat [57,58].

In fresh cheeses, especially in Mozzarella cheese stored in the governing liquid, the growth of *Pseudomonas* and other psychrotrophic commensal bacteria are not counteracted by any inhibiting factor; consequently, their proteases have a dramatic impact on protein structure and quality of these cheeses during cold storage [31,59]. In these conditions, the outer part of high moisture Mozzarella cheese inoculated with a spoilage *P. fluorescens* strain showed a quick hydrolysis of *α*- and *β*-caseins and the increase of free amino acid content in governing liquid [60]. Casein loss results in the wrinkling of the Mozzarella skin and in the weakening of its structure, thus the cheese slowly collapses in on itself [31].

The high content of pseudomonads in raw milk and its late pasteurization before production of pickled cheeses, such as Domiati, Feta and Turkish White cheeses, result in huge loss of protein in the brine during storage [50,61]. In turn, catabolism of free amino acids by *Pseudomonas* strains associated with the dairy environment could fuel several metabolic pathways, among which the production of volatile compounds through transaminases and other hydrolases is detrimental for the quality and shelf-life of fresh cheese [62]. Psychrotrophic pseudomonads also synthesize lipases causing no less serious damage than those produced by proteases [63]; the lipases of psychrotrophic pseudomonads are more active at 4–7 °C than lipases from mesophilic microorganisms and, similar to pseudomonads proteases, show a high stability at temperatures of pasteurization and UHT treatments. Based on these characteristics, the activity of lipases, regardless of the producing strains, can severely damage the quality and shelf-life of especially raw milk as well as high-fat content dairy products during their storage at low temperatures. In fact, they catalyse the hydrolysis of triglycerides to free fatty acids and glycerol [64]. Depending on the length of the fatty acids chain, pseudomonad lipases can lead to the release of C4 and C8 fatty acids associated with rancid flavour and odours; in contrast, free medium-chain fatty acids (C10-C12) produce a soapy flavour. In milk, lipases produced by *P. fragi* can create strawberry-like odour due to ethyl butyrate and ethyl hexanoate esters [62,65].

Butter is one of the dairy products most sensitive to the activity of lipases and phospholipases by psychrotrophic pseudomonads (*P. putrefaciens*, *P. nigrificans*, *P. fragi*, and *P. fluorescens*) [61], which can cause a colour change on the surface (surface stain) and rancidity: *P. putrefaciens* and *P. nigrificans* are able to grow on the surface of butter and produce a black discoloration and a putrid odour within 7–10 days of storage at refrigeration temperature [42]; a skunk-like smell is also developed in butter by *P. mephitica* [66], while *P. fragi* and, rarely, *P. fluorescens* can turn the butter rancid [48].

### 2.2. Discoloration as a Spoilage Trait

Among all the spoilage defects recorded on cold-stored dairy products, the one concerning discoloration is certainly the most studied in the last decade. Indeed, several cases of anomalous discoloration on Mozzarella cheese were due to the contamination by *P. putida* (reddish discoloration [67]), *P. fluorescens* biovar IV and *P. libanensis* (bluish discoloration [40]), *P. gessardii* (yellow and purple spots [40]), and *P. fluorescens* (greenish and fluorescent discoloration [68]), thanks to the production of different pigments (pyoverdine, pyocyanin, pyorubin, and pyomelanin [69]).

In 2010, starting from the European outbreak reported by RASFF’s (Rapid Alert System for Food and Feed), alerting to hundreds of different Mozzarella cheese lots with blue discoloration [70], the concern related to the risk of coloured taints on cheese surface has grown in importance, mostly for dairy companies that need to prevent or trace these spoilage events [71,72]. A method to screen and counteract the synthesis of blue-pigmenting strains was developed by Caputo et al. [60]; the authors also identified the strain (*P. lactis* ITEM17298) and pigment (leucoindigoidine that oxides to blue indigoidine) responsible for the cheese defects [60,73,74]. The work of Caputo and colleagues [60] also proved that the entry into the production cycle of pigmenting pseudomonads leads to the appearance of anomalous taints on Mozzarella cheese even after 2 days of storage. The spoiled product was immediately withdrawn from the market in accordance with current legislation, since it negatively impacts the consumer’s choice and compliance with the original characteristics of the product [75,76].

In milk and fresh cheeses, pseudomonads synthesize and secrete siderophores to overcome iron starvation; these molecules (pyoverdine, pyochelin, pseudomonine, quinolobactin, etc.) work as low-molecular-weight iron chelators causing the appearance of diffusible yellow greenish or fluorescent pigmentation in milk and fresh dairy products [68].

## 3. Biofilm by Food-Related Psychrotrophic Pseudomonads and Its Impact on Dairy Industry

During the preparation, processing of food, and preservation, microorganisms encounter a series of stresses, such as heat, cold, salt, acid, and preservatives; the ability to grow as biofilm—surface-attached communities, embedded in a self-produced extracellular matrix composed of exopolysaccharides (EPS), DNA, and other components—is an important adaptation and survival strategy commonly employed by bacteria. Biofilm lifestyle and associated behavioural changes under unfavourable growth conditions are critical for triggering specific metabolic activities, including spoilage pathways, also needed to maintain the living state of the cells. Single- or multi-species biofilms count 10^8^ to 10^11^ cells/g wet weight [77] and colonize biotic or abiotic surfaces, through different motility systems.

*Pseudomonas* spp. are the most common contaminating strains in dairy manufacturing plants; recent studies demonstrated that ca. 90% of *P. fluorescens* originating from dairy products and dairy plants were able to form biofilm at a wide temperature range (10–30 °C) and within a few hours [8,78]. Table 2 reports a list of *Pseudomonas* spp. isolated from dairy plants and products and the experimental conditions that were assayed to check their biofilm-forming ability. *Pseudomonas* spp. able to produce biofilm were isolated from spoiled foods, food-contact surfaces, and food processing lines [9,78], where they represent one of the major challenges and concerns. The high number of species and their adaptability to grow in a sessile state is shown under several factors (surface characteristics, motility elements, and Ca^2+^ amount [79]) and environmental conditions (low temperature and low concentration of nutrients, Table 2). Among the assayed factors, the combination of longer storage time and lower temperature represents a selective advantage for psychrotrophic pseudomonads that contaminate milk via biofilms previously formed in milk tanks, contaminated water, or soil. Several studies have demonstrated that the major sources of contaminated milk and milk products derive from improperly cleaned/sanitized equipment [80,81]; for example, pasteurized milk can be contaminated by psychrotrophic pseudomonads in the filling machine during the filling operation, which happens when both biofilm penetrates the filler of the vacuum or bulk tanks and disinfection procedures are inadequate or absent [79]. Sources of contamination are also represented by industrial surfaces such as stainless steel, aluminium, glass, PTFE, and Teflon seals [79]; biofilms on plate heat exchangers and pipelines can also cause metal corrosion in pipelines and tanks and reduce the heat transfer efficacy [82]. In order to lower biofilm deposition and thus reducing the undesirable economic and technical issues, modifications of surface hydrophobicity and the use of antimicrobial coatings on plate heat exchangers have been recently developed [83].

Finally, biofilms can contaminate dairy products during packaging and market distribution, adhering to the packaging materials used for dairy products (polyethylene-PET, wood, paper glass, plastic, and metal) [84,85]. In the case of the Mozzarella cheese, instead, *Pseudomonas* spp. biofilms have arisen from the tap water forming the preserving liquid (water, brine, salts, and whey) used to extend a Mozzarella cheese’s shelf-life [39,60]. Some of these bacteria are able to form biofilm under different environmental conditions; however, the highest amounts are reported under low temperature [9]. Under low-temperature storage, pathways associated with this lifestyle also caused anomalous discolorations of Mozzarella cheese.

Cleaning and sanitizing procedures currently in use are often ineffective in removing biofilm. Due to the production of EPS, indeed, biofilms become 10–1000 times more resistant to the antimicrobial agents [86]. Although this aspect is of crucial importance for the determination of the Critical Control Points of Contamination (HACCP), bacterial biofilms are not directly mentioned in the in-use guidelines on food processing facilities. The absence of clear rules and sanitisation actions taking into account biofilm occurrence is a serious gap considering that cross-contamination by a wide range of pathogens is very common [87].

**Table 2:** List of biofilm-forming *Pseudomonas* spp. isolated from dairy plants and products.

*Pseudomonas* Species	Sources of Isolation	Experimental Conditions Assayed for Biofilm Formation	References
*P. fluorescens*	*Burrata*, Butter Curd Cheese, Mozzarella cheese, Milk *Scamorza*, Brine Ice machine, Mozzarella manufacturing plant.	TSB supplemented with 0.2% of glucose, 10 °C at least 48 h of incubation or 30 °C at least 24 h of incubation	[8,78]
	Spoiled milk	TSB, 25 °C, 16 h and exposure for 5 min to 100 ppm chlorine dioxide	[88]
	Floor, drains, pipes, and valves of different processing equipment (raw milk cooling tank, milk separator, skim tank, cream tank, homogenizer, pasteurization vat, milk storage vats, cheese vat, cheese ripening room, and packaging area)	TSB and citrate minimal medium at 4 °C; citrate minimal medium at 30 °C for 48 h	[82]
	Overhead pipe filler in a dairy processing plant	Diluted nutrient medium consisting of 0.05 g/L glucose, 0.025 g/L peptone and 0.0125 g/L yeast extract in 0.02 M phosphate buffer (pH 7), in a flow cell reactor system for 7 days	[89]
	Fresh cheeses	M63 15 °C and 30 °C for 72 h	[9]
	Raw milk	Stainless steel coupons immersed in TSB or skimmed milk at 7 °C for 7 days.	[43]
	Raw milk, pasteurised milk, curd, whey, cheeses environmental surfaces (collected after routine cleaning process) and environmental air from 8 different areas of a dairy industry	On stainless steel coupons immersed in Ultra-high temperature whole milk at exposure temperatures of 7, 13, 27, 41, and 47 °C and contact times of 0, 1.2, 4, 6.8, and 8 days.	[90]
	Mozzarella cheese	Ricotta-based medium 12 °C for 168 h;	[91]
	Dairy product	Pipes filled with skimmed milk diluted 1/10 at 20 °C at 8 rpm	[92]
	Dairy wastewater	TSB (3 g/L) + Ca^2+^ (0.1–1 M) at 30 °C, 150 rpm, overnight 18 h	[93]
*P. putida*	Dairy products	M63 at 15 and 30 °C for 72 h	[94]
	Mozzarella cheese, curd	TSB supplemented with 0.2% of glucose, 10 °C at least 48 h of incubation and 30 °C at least 24 h of incubation;	[8]
	Milk processing line (e.g., balance tank)	BHI, 22 °C and 30 °C, 70 rpm.	[95]
	Dairy plants	Native or modified-surface plate heat exchanger during the pasteurization of raw milk for 17 h.	[83]
*P. granadensis*	Mozzarella cheese	TSB supplemented with 0.2% of glucose, 10 °C at least 48 h of incubation and 30 °C at least 24 h of incubation.	[8]
*P. brenneri*			
*P. brenneri*	Raw milk	TSB, 7 °C for 7 days and stainless-steel coupons immersed in TSB or skimmed milk at 7 °C for 7 days.	[30]
*P. koreensis*	Mozzarella cheese, brine	TSB, 7 °C for 7 days and stainless-steel coupons immersed in TSB or skimmed milk at 7 °C for 7 days.	[8]
	Raw milk	TSB, 7 °C for 7 days	[30]
*P. veronii*	Raw milk	Stainless steel surface immersed in TSB or skimmed milk at 7 °C for 96 h.	[96]
		TSB, 7 °C for 7 days	[30]
*P. fragi*	Stainless-steel surfaces of raw milk tankers floor drains (cooling chamber, and cutting, washing and processing areas of the plant)	Stainless steel surface of an inadequately cleaned tanker immersed in a sterile reconstituted skim milk at fluctuating temperature at a wide range of temperature (16–37 °C) for a maximum of 24 h	[97]
	Raw milk	TSB, 7 °C for 7 days and stainless-steel coupons immersed in TSB or skimmed milk at 7 °C for 7 days	[30]
*P. lactis*	Mozzarella cheese	M63 at 15 and 30 °C for 72 h LB at 15 and 30 °C for 48 h	[35]
*P. gessardii*	Mozzarella cheese	M63 at 15 and 30 °C for 72 h	[94]
*P. cedrina*	Raw milk	TSB, 7 °C for 7 days and stainless-steel coupons immersed in TSB or skimmed milk at 7 °C for 7 days	[30]
*P. azotoformans*			
*P. extremorientalis*		TSB, 7 °C for 7 days	[30]
*P. libanensis*		TSB, 7 °C for 7 days and stainless-steel coupons immersed in TSB or skimmed milk at 7 °C for 7 days	[30]
*P. lundensis*			
*P. lurida*			
*P. simiae*		TSB, 7 °C for 7 days and stainless-steel coupons immersed in TSB or skimmed milk at 7 °C from 96 to 7 days	[30]
*P. yamanorum*		TSB, 7 °C for 7 days and stainless-steel coupons immersed in TSB or skimmed milk at 7 °C for 7 days	[30]
*P. rhodesia*			
*P. rhodesia*	Milk processing line (e.g., balance tank)	Native or modified-surface plate heat exchanger during the pasteurization of raw milk for 17 h	[83]
*P. chlororaphis*			
*P. mucidolens*	Floor drains (cooling chamber, and cutting, washing and processing areas of the plant)	nr	[98]
*P. vancouverensis*			

TSB: Tryptone Soya Broth; M63: Minimal Broth M63; BHI: Brain Infusion Heart; LB: Luria Bertani; nr: not reported.

Recently, the risk that non-pathogenic pseudomonad strains might cause bacteraemia in humans has been highlighted [99]; pathogenic traits of pseudomonads were attributed to several genes expressed under both optimal and refrigerated growth conditions [73]. In addition, pseudomonads can also live as multispecies biofilms, including foodborne bacteria favouring their transmission to the environment and humans; *Listeria monocytogenes* and *Pseudomonas* spp. are frequently co-occurring in the dairy industry [91]. *P. fragi* and *P. fluorescens* have been shown to enhance the attachment to glass surfaces of *L. monocytogenes* and *Aeromonas hydrophila,* respectively (cooperative interaction [30,100]). On the contrary, the growth of *Bacillus cereus* decreases its load when co-existing with *P. fluorescens*, indicating a strong competition [101].

Besides the pathogenic strains, cases of commensalism were reported in *Lactococcus* and *Pseudomonas* mixed biofilms; it has been demonstrated that *P. fluorescens* enhanced lactococcal strains attachment by providing a quickly developed biofilm matrix that hosts them. Meanwhile, the fast growth of *P. fluorescens* likely consumed much of the available oxygen inside the biofilms. The resulting anaerobic conditions may stimulate the growth of the lactococcal strain; the increase in the cell number of both species within biofilms may not necessarily have a significant effect on planktonic counts or milk pH during cold storage [102].

The coexistence of pseudomonads and pathogenic bacteria in biofilm increases the risk related to their spread since biofilms favour persistence in the environment and resistance to common sanitizers and antimicrobials [103]; for example, under dual-species conditions, the simultaneous presence of *L. monocytogenes* strongly increased the resistance of *P. putida* biofilm cells to benzalkonium chloride [104].

The contribution of a single species or mixed biofilms to antibiotic resistance spread along the food chain should be also taken into account [86,94] and increases the need for providing an updated HACCP system evaluating biofilm risk in dairy environments.

## 4. Quorum Sensing in *Pseudomonas* spp.

Biofilms are regulated by a complex cell–cell communication system, well known as *quorum sensing*; this mechanism acts through the secretion and detection of autoinducer molecules, which accumulate in a cell density-dependent manner: when the autoinducer concentrations reach a threshold level, cells activate several regulators and metabolic pathways, allowing them to modulate specific behaviours whose efficacy and fitness benefits depend upon the presence, or absence, of other cells [105]. The QS system is involved in all phases of biofilm formation (attachment, microcolony formation, maturation, and detachment) by fitting the nutritional demands and resources available; it regulates biosurfactant and antibiotic synthesis, pathogenesis, the expression of virulence factors, efflux pumps, enzymes (proteases, lipases, chitinases, and pectinases), and the production of other metabolites (pigments, pyoverdines) also correlated with spoilage behaviour [73,105]. QS also modulates biofilm detachment: when cell densities become high, and the nutrient concentrations become limited inside biofilms, QS activates the release of bacterial cells from biofilms into the environment, favouring the colonization of new surfaces and reinitiating biofilm development.

While *P. aeruginosa* QS is well understood, still little is known about the QS systems of other members of the genus *Pseudomonas*. Therefore, before describing QS-regulated spoilage traits, a summary of the QS systems in the frequently occurring *Pseudomonas* species in the dairy environment and products are reported below.

### 4.1. P. fluorescens

In several *P. fluorescens* strains, QS systems based on N-acyl-homoserine lactones (AHLs) signalling molecules have been identified. AHLs consist of a fatty acyl chain and a lactonized homoserine; their structures vary in the length (4–18 carbon acyl chain), saturation state of the acyl side chain, and the substitution on the third carbon in the acyl chain [106]. The structures of homoserine lactone molecules (HLSs) produced by *P. fluorescens* were reported in several works: Liu et al. [57] found that *P. fluorescens* strain 395 produced C4-HSL and 3OC8-HSL, while *P. fluorescens* 07 and *P. fluorescens* UK4 produced both short-chain and long-chain AHLs [107,108]. In contrast, AHLs were lacking in two strains of *P. fluorescens* isolated from refrigerated raw milk [109].

*P. fluorescens* mainly uses the LuxI/R type QS system; AHL-dependent QS was synthesized by the LuxI homologue and detected by LuxR-type transcriptional regulators [110]. AHLs are synthesized by the autoinducer synthase LuxI from the *S*-adenosyl-l-methionine (SAM) and acylated acyl-carrier protein (acyl-ACP), with the release of holo-ACP and 5′-methylthioadenosine (MTA) as by-products; signals freely diffuse through the cell membrane and when the cell density is high and LuxR binds its cognate autoinducer. Then, the LuxR autoinducer complex binds at the target gene promoters and activates specific pathways, such as biofilm formation, motility, and enzyme production; these latter can be released in high concentrations by the attached cells, leading to food spoilage. *Lux*R/*lux*I homologues in *P. fluorescens* are *phz*I/*phz*R [111] and *mpu*I-*mpu*R [112]. These latter regulate the expression of the mupirocin biosynthetic gene cluster and activates the biosensor *Chromobacterium violaceum* CV026 [112]; the synthesized AHLs were N-(3-hydroxy tetradecenoyl)-homoserine lactone (3-OH-C14:1-HSL), N-decanoyl--homoserine lactone (DHL), and N-hexanoyl-homoserine lactone (C6-HSL [113]).

*P. fluorescens* 2P24 also had a LuxI/R-type QS system, named the PcoIR system, responsible for the production of the AHLs 3-oxo-C6-HSL and 3-oxo-C8-HSL; deletion of *pco*I in the mutant strain caused defective in biofilm formation, colonization, and biocontrol ability [114].

*Hdt*S and *pso*R are part of another QS system discovered in *P. fluorescens* [115,116]; *hdt*S is responsible for a novel AHL synthase producing *N*-(3-hydroxy-7-*cis*-tetradecenoyl) homoserine lactone (3-OH-C14:1-AHL), DHL, and a C6-AHL [115]; however, less is known about the catalytic mechanism of this synthase [115]. In contrast, PsoR (also named LuxR solos) contains an ABD (N-terminal) and DNA-binding HTH C-terminal domain but lacks their cognate LuxI; it has been shown to respond to exogenous and endogenous AHLs produced by neighbouring cells as well as to other molecules/signals [116].

QS genes are in turn regulated by positive and negative regulatory elements that include some two-component regulatory systems (Gac/Rsm signal transduction pathway; [117]: the stationary phase sigma factor RpoS, and the TetR-family member RsaL [118]). The roles of the *rpo*S gene and the GacS/GacA two-component system in the regulation of QS were investigated in *P. fluorescens* 2P24 by Yan et al. [119]. RpoS is a transcriptional regulator induced when bacterial growth transits from the exponential phase to the stationary phase; being a sigma factor, it facilitates the activity of RNA polymerase at a set of defined promoters. In addition to the modulation of extracellular acylated homoserine lactone (AHL) levels (such as C4-HSL, C6-HSL, C8-HSL, C10-HSL, C12-HSL, and C14-HSL [120]), Yan et al. [119] reported a negative feedback relationship between RpoS and the Gac system, a general regulator in the transition from exponential to stationary growth phase conditions. The authors provided the genetic evidence demonstrating that in *P. fluorescens* 2P24 the GacS/GacA two-component system plays a positive role in the transcriptional expression of *pco*I, whilst RpoS repressed the Gac system, acting as negative regulator of *pco*I transcription.

The c-di-GMP signalling cascade is another strategy used by *Pseudomonas* spp. to sense changes in population density and local environmental conditions. Similar to QS, it regulates many bacterial phenotypes and is of key importance for driving the lifestyle switch from fluctuating and lonely cells to sessile communities. In *P. fluorescens* 2P24 the production of the QS signals AHLs was negatively regulated by cyclic-dimeric guanosine monophosphate (c-di-GMP) [121]; c-di-GMP inhibits *pco*I through the expression of *rsm*A and *rsm*E genes, and the latter are inactivated by small noncoding RsmZ such as RNA regulated by GacS/GacA. Similar to its well-established function in biological control through *pco*I, c-di-GMP positively regulates biofilm formation and represses motility in *P. fluorescens* 2P24 [121].

### 4.2. P. lactis

*Pseudomonas lactis* and *P. paralactis*, firstly isolated and identified from bovine raw milk by von Neubeck et al. [122], are described as fluorescent on King B agar, motile, catalase- and oxidase-positive, and able to grow across a wide range of temperature (4–35 °C) and pH (5–8); lipolysis on tributyrin agar and proteolysis using skimmed milk agar were registered at both 25 and 4 °C. Recently, *P. lactis* ITEM 17298 and ITEM 17299 were also found responsible for the blue discoloration of Mozzarella cheese. As a novel species, little information is reported regarding the QS by these strains. This species harbours genetic determinants for Las, LuxR, and RhlI (synonymous BjaI) required for the production of *N*-butanoyl-L-homoserine lactone and *N*-hexanoyl-L-homoserine lactone [35]. In *P. aeruginosa las* and *rhl* QS systems operate in a hierarchy wherein the *N*-3-oxododecanoyl-homoserine lactone, a “gatekeeping” signalling molecule of the *las* system, regulates the *rhl* system at the transcriptional and posttranslational levels [123]. By the activation of a cascade of signals, including the RsmA/RsmZ, the two-component system GacA/GacS, responsible for sensing and responding to environmental stimuli, positively controls the QS operon comprising *las* and *rhl*; in *P. lactis* ITEM 17298 GacA/GacS were described as BarA/UvrY [9,35].

Other QS regulators, such as the acyl-homoserine-lactone synthase EsaR, and the HTH-type QS-dependent transcriptional regulator RpaR, responding to the autoinducer 4-coumaroyl-homoserine lactone, were identified in *P. lactis* isolated from fresh cheeses [9,35]. In *P. aeruginosa* EsaR is an unusual example, as it binds the target DNA in the absence of a signal and serves as a repressor of QS [124].

The LuxR-type regulator (RpaR) was firstly identified as a novel HSL-type QS signal in the Gram-negative *Rhodopseudomonas palustris*; in this species, the *p*-coumaroyl side chain was putatively derived from the exogenous plant metabolite *p*-coumarate and influenced the expression of at least 17 genes also involved in chemotaxis and iron metabolism [125]. Pseudomonads are widely spread in the environment; recently, *P. lactis* was also identified as a foliage endophyte, strongly antagonistic to the boxwood blight pathogen, opening up new avenues for sustainably and effectively controlling its pathogenicity [126]. This evidence may explain the identification of RpaR in *P. lactis*, which would have been diffused from the environment to the dairy products. However, the expression of *rpa*R/*rpa*I in cheeseborne strains and the activation of related pathways in a manner similar to *R. palustris* need to be demonstrated by appropriate experimental trials.

As reported for other bacteria, QS signals are degraded via the acylase mechanism; this activity occurs to sustain energy metabolism through the use of long-chain acyl-HSL (≥8 carbons in the acyl chain: e.g., 3OC12HSL) as the sources of carbon [123]. In *P. aeruginosa* PAO1 and closely related pseudomonads isolated from soil, *pvd*Q and *qui*P genes are involved in QS degradation; *P. lactis* ITEM 17298 harboured both of them, and QuiP was repressed under cold-storage conditions [9]; although the nature of its role in the AHL degradation process still remains elusive, as reported for *P. aeruginosa,* the expression of proteins exhibiting signal decay activities seems not to interfere with cell–cell communications but favouring a balance and modulation of the QS signalling cascade [123]; indeed, different ratios of their long- and short-chain acyl-HSL occur when grown in biofilm or in planktonic states [123]. Since biofilm biomass by *P. lactis* ITEM 17298 increased under cold-storage conditions, we speculate that, in this growth condition, the repression of QuiP could favour the cell–cell communication on the basis of this cellular lifestyle.

### 4.3. P. putida

Similar to *P. fluorescens*, *P. putida* represents the main pseudomonads species found in the dairy chain and responsible for spoilage, even in processed products [32]; however, knowledge on QS by this species have been reported only for strains isolated from vegetable sources. AHL production and response in *P. putida* occurs via the PpuR/RsaL/PpuI QS system, which shows a high homology to the LasR/RsaL/LasI system of *P. aeruginosa* involved in biofilm formation [127]; both systems employ 3-oxo-C12-HSL, 3-oxo-C10-HSL, and, as minor products, 3-oxo-C8- and 3-oxo-C6-HSL as signal molecules. The sequence coding for the homologue of RsaL of *P. aeruginosa* is located in the intergenic region between *ppu*I and *ppu*R and encoded a 76-amino-acid protein of 8.6 kDa; RsaL and Lon protease acts as repressors of AHL production, ensuring the correct timing of the response [128]. Upstream of *ppu*R, the designated gene *ppu*A was expressed in an AHL-dependent manner and showed an involvement in biofilm maturation; by employing a proteomic approach, Sauer and Camper [129] suggested that QS in *P. putida* did not play a role in the initial attachment process but in the late stages of biofilm development.

As reported for other *Pseudomonas* species, in *P. putida* there is a link between the PpuR/RsaL/PpuI system with GacA and the stress sigma factor RpoS, demonstrating that the three global regulatory controls are intimately connected in response to the stationary phase. AHL-dependent QS has a positive effect on *rpo*S transcription; in turn, RpoS has a negative effect on QS, while GacA has a positive effect [130].

Besides biofilm formation, PpuR/RsaL/PpuI also regulates activities such as proteolysis [127] and the production of cyclic lipopeptides (putisolvin I and II [131]); these biosurfactants could stimulate the colonization of more favourable niches, enhancing the competitiveness (fitness) and pollutant-degradation capabilities [131].

### 4.4. P. fragi and P. gessardii

Although *P. fragi* and *P. gessardii* spoilers are frequently isolated both in dairy products [31], meat, and vegetables [132], studies investigating QS and related spoilage pathways in these species are scarce or completely absent [133,134]. Thus, in order to shed a light on the putative QS systems regulating metabolic activities in these species, we analysed the genome of *P. fragi* P121 and *P. gessardii* BS2982; these were selected among the deposited available genomes for the completeness of the assembly or for being the representative strain, respectively.

In *P. gessardii* we found genetic determinants for LasR/LasI (Accession Number SDQ93893.1/SDQ93867.1) and phenazine biosynthesis (PhnA/PhnB; Accession Number SDR24489.1/SDR24512.1); in *P. aeruginosa* the anthranilate synthase PhnAB is not directly involved in phenazine biosynthesis but rather produces anthranilate for the generation of the *Pseudomonas* quinolone signal (PQS), and in turn the regulator required for phenazine biosynthesis; in these strains, indeed, the PQS synthetic cluster has been revealed to consist of *pqs*ABCDE, *phn*AB, and *pqs*H [135]. However, the operon *pqs*ABCDE and *pqs*H were not found in *P. gessardii*.

Ferrocino et al. [133] reported that 72 isolates of *P. fragi* were not able to produce HSL; this finding suggested the absence of genes related to HLS synthesis, although genomes were not investigated by the authors. Based on this study, we analysed the *P. fragi* P121 genome and this hypothesis was confirmed. In addition to this, *P. fragi* P121 did not even harbour a *lux*S gene, responsible for the synthesis of furanosyl borate diesters (type II autoinducers; AI-2) that were instead detected by Ferrocino et al. [133].

Further information related to QS and regulated metabolic activities of these species should be carried out since they were often found among biofilm-producing species and spoilage bacteria in cold-stored foods.

## 5. QS-Regulated Spoilage Traits in Dairy-Borne *Pseudomonas* spp.

**Figure 2:**
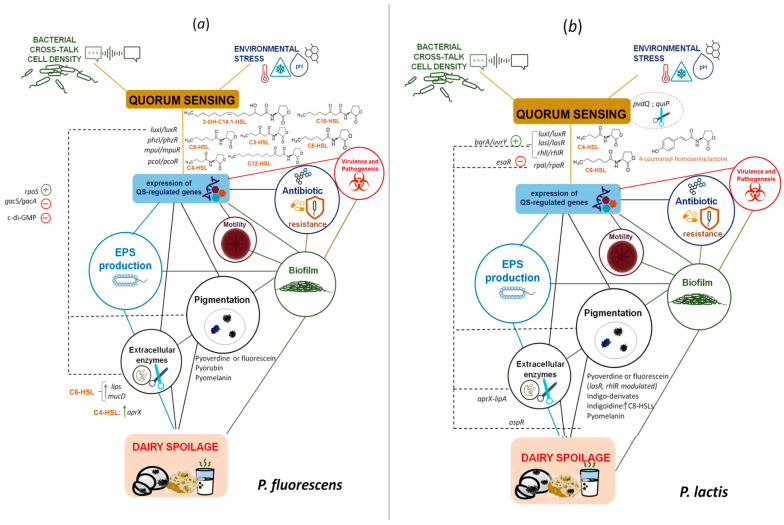
Major spoilage traits regulated by *quorum sensing* systems reported for (**a**) *P. fluorescens* and (**b**) *P. lactis*. N-(3-Hydroxybutanoyl)-L-homoserine lactone (C4-HSL); N-hexanoyl-homoserine lactone (C6-HSL); N-3-oxo-octanoyl-L-Homoserine lactone (C8-HSL); N-decanoylhomoserine lactone (C10-AHL); N-dodecanoyl-L-Homoserine lactone (C12-HSL); N-(3-hydroxy-7-cis-tetradecenoyl) homoserine lactone (3-OH-C14:1-AHL); 3′,5′ Cyclic diguanylic acid (c-di-GMP); two-component system GacA/two-component system GacS (*gac*A/*gac*S); RNA polymerase sigma factor RpoS (RpoS); acyl-homoserine-lactone synthase/transcriptional activator protein PhzR (*phz*I/*phz*R); acyl homoserine lactone synthase/LuxR family transcriptional regulator (*lux*I/*lux*R); acyl-homoserine-lactone synthase/transcriptional regulatory protein PcoR (*pco*I/*pco*R); triacylglycerol lipase (*lip*S); periplasmic serine endoprotease DegP-like (*muc*D); Metalloprotease AprX (*apr*X); two-componenent system BarA/UvrY-regulatory subunit (*bar*A/*uvr*Y); acyl-homoserine-lactone synthase esaR (*esa*R); cyl-homoserine-lactone synthase/transcriptional activator protein LasR (*las*I/*las*R); acyl-homoserine-lactone synthase/regulatory protein RhlR (*rhl*I/*rhl*R); lipoyl synthase (*lip*A); exopolysaccharide (EPS).

The advances in technological methodologies used to study the evolution of QS systems in the different growth conditions and time of incubation allowed researchers to shed a light on several spoilage activities in QS-regulated biofilms: several AHLs have been indeed detected in foods spoiled by psychrotrophic bacteria; in addition, AHLs concentration was proved to increase with the degree of food spoilage [13,14]. A deep analysis of the QS spoilage-related genes and pathways is reported below and for *P. fluorescens* and *P. lactis* also displayed in Figure 2a,b.

### 5.1. Proteases, Lipases and Phospholipases

Hydrolytic enzymes are usually produced in the late exponential/early stationary growth phase when the organisms have reached comparatively high cell densities (>10^6^ cfu/mL) and can be released from the biofilms into the milk without bacterial detachment [97].

Recently, RNA-sequencing was employed to explore the involvement of QS in dairy spoilage caused by *P. azotoformans*. Fifteen pathways among those analysed were significantly affected by C6-HSL [13]; these latter included cell division, energy metabolism, and nutrient uptake, which are crucial for bacterial survival and adaptation in the food processing environment. Some of the genes upregulated by C6-HSL were elongation factors and *sec/yaj*C/*yid*C, responsible for protein synthesis and secretion, respectively; the type VI secretion system T4SS used for the transport of proteins and DNA across the cell envelope; and *lip*S, *muc*D, and the probable periplasmic serine endoprotease DegP-like (PputW619_1070). In Gram-negative bacteria (e.g., *E. coli*), DegP serine endoproteases are involved in regulated intramembrane proteolysis (RIP) cleaving transmembrane proteins to liberate a cytosolic domain of proteins able to modify gene transcription, such as the two-component regulatory system CpxA/CpxR, which responds to envelope stress response [136].

In milk, the AHLs signal molecules (C4-HSL and 3OC8-HSL) from *Pseudomonas* species, such as *P. azotoformans*, were found to change their particle size distribution, and lead to the destabilization of UHT milk during storage; this feature, as well as off taste and off odour, was putatively attributed to the production of QS-regulated heat-resistant proteases and lipases produced by psychrotrophic bacteria [13,137]. Indeed, AHLs and other AHL-related products from *Pseudomonas* species have been shown to increase the activity of the *apr*X promoter; the activity was instead repressed in presence of the enzyme AHL lactonase hydrolysing AHLs [35,57]. Post-transcriptional regulation, under the control of the GacS/GacA two-component regulatory system, was also suggested to take place in the expression and secretion of protease in this system [57]. AprX is mainly secreted by the species *P. fluorescens,* but it has also been detected in various other species found in raw milk such as *P. fragi, P. tolaasii, P. rhodesiae, P. gessardii, P. proteolytica, P. brenneri,* or *P. chlororaphis*. Recently, the genomic analysis of three *P. lactis* individuals isolated from dairy products identified several protease genes (*apr*A, *prs*DE, * prt*AB), positioned in a QS-regulated operon previously associated with milk spoilage by *P. fluorescens* [73]; *apr*A was described as 98% similar to the peptidase *apr*X [138]. AprA and AprX hydrolyse the four types of casein (αs1, αs2, β, and κ) with a large activity spectrum, and generally exhibit activity in a large range of pH (4.5–10), with optimum activity between 7.5 and 9, as well as across temperatures (0–55 °C), with optimal activity between 37 and 47 °C [139].

Liu et al. [108] reported that the sigma factor RpoS, a positive regulator of two AHL synthase genes and three coding for LuxR-like transcription factors, is a key regulator of spoilage activity by *P. fluorescens*. Under food-processing conditions (exposure to heat, cold, salt, acid, and preservatives), it positively regulates the extracellular protease activities and the total volatile basic nitrogen production; thus, its monitoring during food processing and storage is considered a useful strategy to ensure the quality and safety of the final food.

In several species of *Pseudomonas* spp. (e.g., *P. fluorescens* and *P. psychrophila*) the production of lipases were regulated by C4-HSLs; these latter increased the proteases and lipases production in pasteurized milk after incubation at 48 °C for 18 h and caused milk spoilage [11]; moreover, the occurrence of AHLs in heat-treated milk demonstrated that they retained all or at least part of their activities, including the modulation of *Pseudomonas* spp. growth [140].

Few studies report the regulation of lipase genes and include *P. aeruginosa;* in this strain the expression of the lipase genes was controlled by the RhlR/I system and Gac system [141]. In *P. lactis* and *P. fluorescens* [35,138], isolated by dairy products, the activity of lipase included in *apr*X-*lip*A operon were by the homologue of the *E. coli env*Z-*omp*R affected by environmental osmolarity and regulating biofilm formation [142].

The phospholipase C of *P. fluorescens* is a heat-stable enzyme, presenting high residual activity after pasteurization and UHT treatment; it is able to hydrolyse intact milk fat globules by increasing the lipase activity. Similar to lipases and proteases, phospholipase C activity is highest in the stationary growth phase and is regulated by the Gac system [143].

### 5.2. Pigments

Dairy products often appear discoloured due to the biosynthesis of pigments (pyoverdine or fluorescein, pyorubin, pyomelanin, pyocyanin, and indigoidine) by some *Pseudomonas* species [94]; pigment synthesis is putatively orchestrated to counteract the increased oxidative stress that pseudomonads undergo at low temperatures [94], as well as to modulate the transition to planktonic to biofilm state, to act as antimicrobials against other microorganisms or as signalling molecules and virulence factors [35].

By combining different omics approaches, it was possible to identify the genomic locus unique to blue pigmenting *Pseudomonas* spp. [9,73]. The “blue branch” of the *P. fluorescens* phylogenetic tree include strains harbouring a genomic locus, indicated by Andreani et al. [72] as the c4_BAR (Contig 4 Blue Accessory Region); this region was described as containing 16 genes (16 Kb), including those coding for the trp accessory genes *trp*D, * trp*F, * trp*A, * and trp*C. Quintieri et al. [73] identified this region also in other pseudomonads; the analysis of the genomic context of the flanking regions suggested this region as a hotspot for genomic island integration. Quintieri’s group revealed the pathway related to the blue indigoidine synthesis by its inhibition in the presence of lactoferrin-derived antibiofilm peptides [9,35]. In Gram-negative bacteria, such as *Roseobacter* spp., indigoidine synthesis was modulated by C8-HSLs [144] through a multi-layered control exercised by a LuxRI-like system integrated with c-di-GMP [145,146]; pigment production was found to confer a competitive advantage to this strain when grown in co-culture with other microorganisms [145].

In addition to leucoindigoidine, the *P. lactis* ITEM 17298 harboured genes associated with the synthesis of the dark pigment pyomelanin from homogentisate (HGA) [35,73]. The metabolic route correlated to pyomelanin synthesis crossed the QS-regulated shikimate pathway, producing chorismate from D-erythrose 4-phosphate, a pentose phosphate intermediate; then, chorismate was converted to tryptophan. This latter pathway was recently associated with indigo derivate pigments by Andreani et al. [72,147].

In *P. aeruginosa*, Lan et al. [146] identified the oxidative stress sensing and response *osp*R (oxidative stress response and pigment production Regulator) gene, which binds to the promoter region of homogentisate 1,2-dioxygenase (*hmg*A) and affects its expression; the *hmg*A gene is involved in pyomelanin production. Orthologues of *osp*R are also present in *P. fluorescens* strains while they are absent in other pseudomonads such as *P. putida*, *P. syringae*, and *P. entomophila*. In addition to protection against oxidative stress, *osp*R plays multiple regulatory roles as a transcriptional regulator of β-lactam-resistant and QS-related genes (e.g., *phz*M, *phz*S) [146].

*Pseudomonas* spp. also produced fluorescent pigments on the cheese surface [148], putatively correlated to the synthesis of fluorescent siderophores, well known as pyoverdines (Pvds) or pseudobactins, which are involved in iron uptake and storage [9]; providing iron to the cell is especially important during the lag phase of growth, when the total siderophore concentration could be low. After binding its specific receptor, iron-bound pyoverdines act as signalling molecules that trigger the expression of several genes, e.g., those involved in the secretion of toxins responsible for virulence in pathogenic strains or for competitive advantage in the presence of other *Pseudomonas* spp. Moreover, pseudomonads pyoverdines can mediate proteases and lipases activities during spoilage [10,149,150].

As described by Machado et al. [139], who reported the inhibition of pyoverdine synthesis and proteases by applying a natural plant extract able to intercept the QS systems (*las* and *rhl*) involved in their expression, the identification and application of QS inhibitors to counteract pseudomonads growth, spoilage, or pathogenesis represents a promising preservation technique to improve the shelf-life of foods.

### 5.3. Off Flavours

Both milk fats and proteins release the elements responsible for the off flavours associated with dairy products. In particular, milk fats and triglycerides release short-chain fatty acids and keto and hydroxy acids that can react, leading to a bitter, soapy taste in fluid milk [64]; in addition, unsaturated fatty acids and phospholipids are substrates for autoxidation reactions. In contrast, proteins are a source of sulphur compounds and amino acids; the first ones are responsible for the cooked flavour in heated milk, whereas the second ones react with reducing sugars through the non-enzymatic browning reaction that produces caramel-like flavours [151]. As discussed above, fats and proteins hydrolysis occur thanks to the activity of protease and lipases, positively affected by QS; the kind and amount of released off-flavour compounds depend on the activated enzyme and substrate.

For example, the presence of exogenous C6-HSL in milk inoculated with *P. azotoformans* increased the lipolysis of fat in milk with a consequent higher content of volatile compounds (acetone, 2-heptanone, 2-butanone, and 2-pentanone) and acids (acetic acid, hexanoic acid, and octanoic acid) [13]. Likewise, hydrophobic peptides, derived by the hydrolysis of casein and responsible for the bitter off flavours, were released by QS-regulated AprX [9,152], as previously described.

Free amino acids are also substrates for the production, by pseudomonads decarboxylases, of biogenic amines (BAs, e.g., monoamines tyramine, histamine, cadaverine, and putrescine), a group of toxic compounds that can negatively affect the sensory properties of dairy products during storage; e.g., cadaverine and histamine impart a putrid and pungent flavour to milk [153]. It has been reported that in *Pseudomonas* spp. some BAs (such as putrescine) act as signalling molecules, triggering the attachment and biofilm formation; they also protect bacteria against radiation and oxidative stress and contribute to the development of antibiotic resistance and pathogenesis [154]. The effect of exogenous C4-HSL, C6-HSL, C8-HSL, C12-HSL, and C14-HSL on total volatile basic nitrogen (TVB-N), which reflect the accumulation of biogenic amines, was demonstrated by Li et al. [14] in aquatic products inoculated with *P. fluorescens*.

## 6. Methodological Advances, Perspectives and Concluding Remarks

The detection of QS signals in spoiled food products paves a new way to study the process of food spoilage. Monitoring AHLs signalling molecules in food samples can be a useful strategy to counteract spoilage events and preserve food quality and safety. To this aim several methodologies have been developed, both to detect and block the AHLs signalling network [155,156].

In the “pre-high-throughput” era the use of an investigating methodology based on individual methodological approaches gave only a partial vision of the complexity underlying the QS mechanisms, often leading to incomplete outcomes. When high-throughput technology started to revolutionize science, omics approaches became the foundation to reveal singular levels of QS systems. The first genetic approach to study biofilm was described in 1999 by O’Toole et al. [157,158] for the screening of biofilm-defective mutants in *P. aeruginosa*, *P. fluorescens*, *Escherichia coli*, *Vibrio cholera*, *Shewanella putrefaciens*, and *Staphylococcus epidermidis*.

The use of genomics, metagenomics and functional genomics has allowed to screen microbial communities or single strains in order to identify the genetic determinants of QS behaviours: since 2005, Williamson et al. [159] developed a metagenomic-based approach for the intracellular screening of QS system, designated as METREX, by which it was possible to capture and sort metagenomic DNA clones that activated QS inducers producing a fluorescent signal detected by biosensors. By the availability of genomic data, furthermore, it was also possible to reconstruct the phylogeny, acquisition, and evolution of single QS genes; e.g., *lux*S, responsible for the production of AI-2, is present ubiquitously across the bacterial domain and found in over half of all sequenced bacterial genomes [160,161]; the congruity between the QS and rRNA trees suggested that QS proteins within the Proteobacteria are of ancient origin [162].

One of the biggest challenges in the identification of signals involved in QS is that their activation is highly dependent on the environmental conditions, the external stress, and the substrate. Different stimuli can trigger specific pathways and signal cascades, thus complicating the identification of global regulators of QS. However, some pathways are shared, such as those involved in EPS biosynthesis [105] or stress-induced genes [163].

To overcome the methodological limitations, in silico studies are designed to model under a wide range of environmental conditions and bacterial population composition the evolution of QS traits. In 2020, Wang et al. [164] used a modelling approach to compare the response of QS systems under largely unknown ecological context or defined ecological challenges, underlying the role of genetic pressures on the shorter-term dynamics of QS. Simulation platforms, such as the Avida [165] and Aevol [166], are increasingly employed to perform in silico evolutionary experiments.

While the detection of QS molecules is largely achieved by biosensors [167] the identification and characterization of QS molecules is mainly assigned to metabolomics studies [168,169]. Furthermore, QS has been extensively studied in relation to clinical environments; however, regarding its involvement in improving bacterial fitness and colonization, as well as its implication on health, the knowledge of this system with respect to food is still limited. In particular, QS-based pathway analysis in relation to dairy spoilage is indeed more recent and the combination of omics methodologies in this field has contributed to describe common features, such as the production of AHL, responsible for milk and dairy spoilage [9,73,170].

The multi-omics approach, which in some cases is also used to validate hypotheses formulated by modelling, confirmed its promising efficacy also in the identification of QS inhibitors to be applied to counteract biofilm formation in several environments.

QS inhibition, also called *quorum quenching* (QQ), includes (a) the enzymatic degradation of signal molecules; (b) the blocking of signal generation; and/or (c) the blocking of signal reception [171]. In this context, food has been used mostly as a source of QSI [12,172], rather than the applicative framework. Despite the natural origin of these molecules making their use in food relatively easy in relation to safety, there are several challenges related to the analysis and the validation of the effects of putative QSI in food, as reviewed by Skandamis and Nychas [173]: the complexity and heterogeneity of the food matrix; the assay used, which could generate different responses in different species although using the same QS system; the importance of the inoculum effect; and the risk of masking of QSI activity in a larger population in relation to cell-to-cell variability.

Few molecules have been identified as QSI against spoilers. Many plant essential oils and their components extracted from oregano, sage, and marjoram have been proved to be effective as antimicrobials and against postharvest decay [174]: essential oils from *Murraya koenigii* and furanones were shown as the most promising QSI, delaying spoilage by psychrotrophic pseudomonads in refrigerated milk [16,17]; an in silico analysis (docking and molecular dynamics simulation) by Gopu et al. [175] demonstrated the quercetin activity against QS-regulated phenotypes of food-borne pathogens, including biofilm formation, EPS production, and flagellar-mediated motility; garlic extract, for example, exerts a strong antagonistic effect on LuxR-based QS; therefore, it has been widely used in the food and flavour industry and might be used as alternative preservatives to prevent or delay food spoilage, or to predict the shelf-life of stored foods [173]; recently, a virtual screening from a food-derived compound database was used by Ding et al. [107] to identify potential QSIs against the strain *P. fluorescens* P07: catechin showed the highest anti-QS activity. Despite the increasing number of QSI recently discovered, their unclear mechanism of action, especially in food matrices, the effectiveness limited toward few species, and the complexity in developing a stable and safe application in the dairy production process make their use still far from implementation.

Food spoilage causes losses of billions of dollars worldwide every year; in Europe, 20% of total production is lost every year, with severe economic and social impacts. This value includes more than 100 tonnes of dairy products that are wasted globally, with almost half lost before they even reach a store. Therefore, in line with official departments and public bodies guidelines, the development of control strategies able to improve the shelf-life of dairy products should be reinforced. The need to increase knowledge on microbiota composition, metabolic pathways, enzymes, and molecules involved in spoilage activity is fundamental to reach this goal. With the evidence of *Pseudomonas* spp. contamination as a major cause of dairy decay, this review has deeply investigated all the well-known issues, focusing for the first time on the role of microbial cross-talk in the evolution of spoilage events. Indeed, recently, molecule signals involved in *quorum sensing* have been detected in spoiled products where they affect microbial biodiversity and metabolic activities; these could be exploited as useful markers to monitor the quality of dairy products under storage and prevent spoilage events. Due its risk for human health, until now, extensive research on QS has been carried out for *P. aeruginosa*, whilst QS systems in other species (such as *P. fluorescens*, *P. fragi*, *P. gessardii*, *P. ludensis*, etc.) responsible for a quickly decay of food products are still poorly explored. It is worthy to highlight also that these latter species, contaminating dairy facilities as biofilm, can favour the spread of pathogenic and MDR strains; this phenomenon is also worsened by the absence of guidelines and Clear Critical Control Points of Contamination (HACCP) for the control of these species in the food chain. Thus, advancements in the knowledge of QS and the regulated metabolic activities represent both a considerable potential to counteract spoilage in dairy cheese and a starting point for searching novel molecular targets in the development of preservation strategies based on natural antimicrobials and QS inhibitors.

## Figures and Tables

**Table 1:** Spoilage traits on dairy products by *Pseudomonas* spp.

Spoilage Traits	Pseudomonas Species	Dairy Product	References
**Proteolysis**			
Milk creaming, sediment formation, gelation, bitterness	*P. fluorescens,* *P. weihenstephanensis,* *P. proteolytica,* *Pseudomonas* spp.	UHT milk	[25,26,27]
	*P. panacis*	Skimmed milk	[28]
	*P. azotoformans*	Raw milk	[29,30]
	*P. gessardii*	Milk	[26,31]
	*P. proteolytica*		
	*P. fluorescens*	Unpasteurized goat milk	[32]
	*Pseudomonas* spp.	Non-bovine raw milk	[33]
Bitterness, Mozzarella skin wrinkling/peeling, cheese softness, sediment formation	*P. lundensis,* *P gessardii*	Mozzarella cheese, skimmed milk	[31]
	*P. fluorescens*	Crescenza cheese	[34]
	*P. fluorescens,* *P. fragi*	Mozzarella cheese	[31]
	*P. fluorescens,* *P. putida*	Cheddar cheese	[25]
**Discoloration**			
Blue	*P. fluorescens,* *P. lactis*	Mozzarella cheese	[8,35]
	*P. fluorescens*	Latin-style fresh cheeses	[36,37]
	*P. carnis,* *Pseudomonas* spp.	Brazilian fresh soft cheese	[38]
	*P. fluorescens*	Mozzarella processing fluids	[39]
Orange or Orange-red-brown	*P. aureofaciens,* *P. gessardii,* *P. putida biovar II*	Mozzarella cheese	[40]
Greenish	*P. fluorescens*		
Fluorescent (yellow-green)	*P. fluorescens,* *P. putida,* *P. palleronii*		
Grayish	*P. azotoformans*	HTST milk	[41]
	*P. fragi*	Milk	[40]
Black	*P. mephitica,* *P. nigrifaciens*	Butter	[42]
**Lipolysis**			
Rancidity off-flavors straw-berry flavor bitterness soapy	*Pseudomonas* spp., *P. fluorescens*	Sterilized milk	[43,44,45]
	*P. fluorescens*	Ripened semi-hard cheese	[46,47]
	*P. fragi,* *P. putrefaciens,* *Pseudomonas* spp.	Cream, butter	[44,48,49]
	*Pseudomonas* spp.	Domiati cheese	[50]
	*Pseudomonas* spp.	Soft cheese	[51]
Modification of rennet coagulation time and curd firmness	*Pseudomonas* spp., *P. fluorescens*	Cheese Cheddar cheese	[44,52]

## Data Availability

Publicly available datasets were analyzed in this study. This data can be found here: https://www.ncbi.nlm.nih.gov/nuccore/FNKR00000000.1; https://www.ncbi.nlm.nih.gov/nuccore/CP013861.1.
